# Run Your 3D Object Detector on NVIDIA Jetson Platforms:A Benchmark Analysis †

**DOI:** 10.3390/s23084005

**Published:** 2023-04-15

**Authors:** Chungjae Choe, Minjae Choe, Sungwook Jung

**Affiliations:** 1 Autonomous IoT Research Center, Korea Electronics Technology Institute, Seongnam 13509, Republic of Korea; cjchoe1@keti.re.kr; 2 Caterpillar Inc., Peoria, IL 61629, USA; minjae.choe@cat.com

**Keywords:** 3D object detection, deep learning, Jetson platforms, benchmark

## Abstract

This paper presents a benchmark analysis of NVIDIA Jetson platforms when operating deep learning-based 3D object detection frameworks. Three-dimensional (3D) object detection could be highly beneficial for the autonomous navigation of robotic platforms, such as autonomous vehicles, robots, and drones. Since the function provides one-shot inference that extracts 3D positions with depth information and the heading direction of neighboring objects, robots can generate a reliable path to navigate without collision. To enable the smooth functioning of 3D object detection, several approaches have been developed to build detectors using deep learning for fast and accurate inference. In this paper, we investigate 3D object detectors and analyze their performance on the NVIDIA Jetson series that contain an onboard graphical processing unit (GPU) for deep learning computation. Since robotic platforms often require real-time control to avoid dynamic obstacles, onboard processing with a built-in computer is an emerging trend. The Jetson series satisfies such requirements with a compact board size and suitable computational performance for autonomous navigation. However, a proper benchmark that analyzes the Jetson for a computationally expensive task, such as point cloud processing, has not yet been extensively studied. In order to examine the Jetson series for such expensive tasks, we tested the performance of all commercially available boards (i.e., Nano, TX2, NX, and AGX) with state-of-the-art 3D object detectors. We also evaluated the effect of the TensorRT library to optimize a deep learning model for faster inference and lower resource utilization on the Jetson platforms. We present benchmark results in terms of three metrics, including detection accuracy, frame per second (FPS), and resource usage with power consumption. From the experiments, we observe that all Jetson boards, on average, consume over 80% of GPU resources. Moreover, TensorRT could remarkably increase inference speed (i.e., four times faster) and reduce the central processing unit (CPU) and memory consumption in half. By analyzing such metrics in detail, we establish research foundations on edge device-based 3D object detection for the efficient operation of various robotic applications.

## 1. Introduction

Deep learning-based 3D object detection is essential for mobile platforms, such as autonomous vehicles, robots, and drones to measure the position and heading direction of neighboring objects quickly and accurately [1]. To enable real-time computation for object detection tasks, an emerging trend is on-device processing that utilizes a built-in computer. As mobile platforms often require real-time control to avoid dynamic objects, on-device processing can enhance smooth navigation without collisions. Recently, several approaches have been developed to build robotic applications on NVIDIA Jetson boards since they offer suitable computational performance with a lightweight size [2]. However, 3D object detection (i.e., deep learning-based point cloud processing) on the Jetson has not been extensively tested in terms of accuracy, inference speed, and resource usage. Therefore, a thorough measurement of the Jetson for deep learning-based 3D object detection is required to provide a feasibility analysis of the boards for robotic applications.

Recent open-source frameworks for deep learning-based 3D object detection have shown promising performance in enabling autonomous driving but require expensive computations. Two-stage detectors [3,4,5] achieve fairly high precision for object detection owing to an extra refinement stage. Although one-stage detectors [6,7,8] have slightly lower precision than two-stage algorithms, they are faster due to their simpler deep learning architecture. Moreover, the latest one-stage detectors [9,10] show advanced detection precision comparable to two-stage algorithms. However, both approaches may not assure smooth operation in resource-constrained devices due to the expensive point cloud computations.

There have been benchmark studies that measure the performance of Jetson platforms. Some studies [11,12,13] test Jetson boards for deep learning applications, including image-based and point cloud-based classifications. However, there is no extensive study that evaluates the Jetson for deep learning-based 3D detection, which is a fundamental function of autonomous driving. Compared to 2D object detection, which mostly depends on the resources of the graphics processing unit (GPU), 3D object detection involves additional point cloud processing and considerably utilizes the entire computing resources. Thus, a suitable benchmark is required to confirm the potential of the Jetson platforms for operating 3D object detectors in real-time.

**Figure 1:**
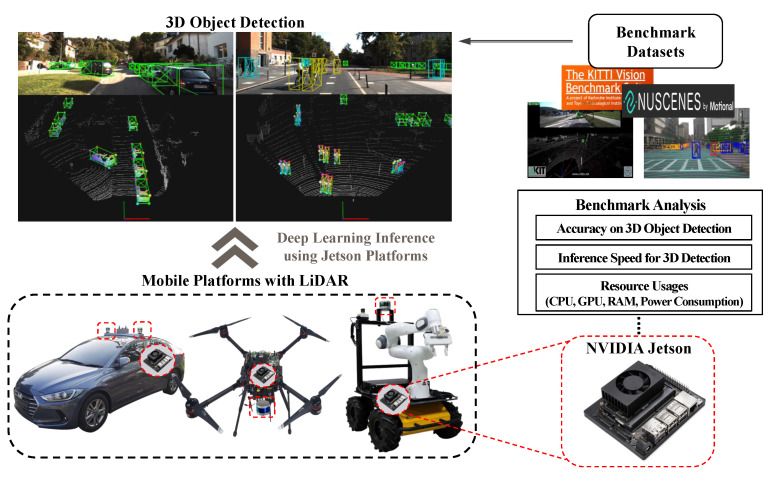
The benchmarking concept of the study. The Jetson platform could be deployed to mobile platforms to run a deep learning-based 3D object detector for autonomous navigation.

In this paper, we present a benchmark analysis of 3D object detection frameworks on NVIDIA Jetson platforms. This study extensively examines the performance of the Jetson when operating open-source 3D object detectors. The benchmarking concept of this paper is shown in Figure 1. To achieve this objective, we tested all commercially available Jetson series, including Nano, TX2, Xavier NX, and AGX Xavier. Moreover, we selected state-of-the-art detectors that provide suitable accuracy and fast inference for enabling autonomous driving. We ran those detectors with the KITTI benchmark dataset [14], which includes point cloud data collected from the Velodyne HDL-64E device and ground truth labels of various objects. We exploited the TensorRT (TRT) library [15] to test an optimization effect for faster inference of Jetson platforms. We investigated the performance of the Jetson in terms of three metrics, i.e., detection accuracy, frame per second (FPS), and resource usage (e.g., central processing unit (CPU), GPU, memory, and power consumption).

The following are the contributions of this work:

- This paper presents extensive benchmarks of the NVIDIA Jetson platforms for a computationally expensive task, which is deep learning-based 3D object detection. Unlike image-based 2D object detection, which mostly depends on GPU resources, the 3D detection task presents a challenge for resource utilization since point cloud processing occupies considerable CPU and memory usage, and neural network processing highly relies on GPU-based computation. Thus, we conducted a thorough analysis of the performance and utilization to provide a guideline for users in choosing an appropriate platform and 3D object detector.
- We evaluated various 3D object detection frameworks with all commercially available Jetson platforms, including the lightest platform (Nano) and the most recent one (NX). We provide various metrics-based results, such as detection accuracy, FPS, and resource usage.
- In addition, we evaluated the effect of the TRT library [15] on optimizing deep learning models to enable fast inference on Jetson platforms. We investigated the impact of an optimization strategy on performance changes with resource usage.

## 2. Related Works

The goal of our study is to benchmark NVIDIA Jetson platforms for the deep learning-based 3D object detection task. We introduce the following two research trends: (i) studies on 3D object detection; (ii) benchmark tests on the Jetson platforms.

### 2.1. Deep Learning-Based 3D Object Detection

Recently, there have been studies focusing on improving the quality of images for object detection [16,17]. However, relying solely on monocular images for 3D object detection may not yield sufficient performance. In this context, point cloud-based 3D object detection plays a crucial role in autonomous driving as it provides pose information about nearby objects, enabling successful navigation without collision. Two types of Light Detection and Ranging (LiDAR)-based 3D object detection schemes are typically used: a two-stage detector and a single-stage detector. The former consists of a region-proposal stage and a classification stage, where the former roughly estimates object locations and the latter refines the predicted bounding box of objects. There have been several studies [3,4,5] that provide high precision of 3D object detection tasks using the two-stage approach. The algorithm presented in [3] employs a well-known convolutional architecture (i.e., PointNet++ [18]) and proposes a bottom-up approach that generates the region-of-interest (ROI) from raw point cloud input. According to a simple abstraction of the raw point cloud, the algorithm [3] could deteriorate when a sparse point cloud is given from occluded or far objects. Moreover, there are voxel-based approaches [4,5] that divide the point cloud input into a voxel space and adopt a 3D convolutional network for an accurate region proposal procedure. These approaches define RoI on the point cloud to extract useful features from the given input during the region proposal step. This RoI-based feature selection can improve recognition results with sparse space, but it still relies on heavy computation. Recently, a novel approach [19] that uses center points of objects with a keypoint detector has shown state-of-the-art performance. Specifically, this method performs better predictions for the pedestrian moving direction since it uses the center points of the object, unlike previous methods [3,4,5] that rely on anchors. While these methods can detect objects with high precision, they may not provide enough FPS for mobile robotic systems due to the complex computations required by the large size of deep learning models.

The single-stage approach, which operates region proposals and classification simultaneously, could be a suitable solution for robotic platforms with resource-constrained devices since it usually runs faster owing to its simpler procedure. In [6], the authors adopted the network structure of You Only Look Once (YOLO) [20] with an additional method for the pre-processing of point clouds. They used the bird’s eye view (BEV) map as input for a neural network and employed an Euler-region-proposal network (E-RPN) to detect the 3D position of objects with a heading direction. The proposed algorithm achieves fast inference but degrades when detecting objects with sparse point clouds, such as distant pedestrians. Another work in [7] modifies the voxel-based approach by replacing normal convolutional layers with sparse convolutional layers that extract useful features from non-empty voxels. The algorithm shows a faster inference speed than VoxelNet [8] by five times. For further improvement in inference speed, PointPillar [21] adopts a novel encoder in which an input point cloud is converted into several pillars. The algorithm achieves impressive speed growth by extracting features of those pillars with 2D convolutional layers.

To enhance the detection accuracy of the single-stage method, some algorithms add post-processing not only to the feature extraction phase. In [9], the authors present advanced post-processing paradigms such as intersect over union (IoU) shaping and IoU-weighted non-maximum suppression (NMS) to attain high performance for car detection. Moreover, a teacher–student paradigm [10] that combines with an orientation-aware distance-IoU loss achieves top accuracy for car detection. The proposed single-stage methods not only provide fast object detection but also show improved detection accuracy. Both the aforementioned one-stage and two-stage detectors have their own advantages, and users need to choose what to use depending on the applications while considering available resources and desired computation speed. Thus, we provide a detailed analysis of these detectors to effectively assist with such a tricky selection.

### 2.2. Applications and Benchmarks of Jetson Series

Jetson platforms have been deployed to robotic tasks owing to their deep learning compatibility [2]. The algorithm presented in [22] provides real-time depth reconstruction (i.e., higher than 15 FPS) where the algorithm operates on Jetson TX2. It uses fully convolutional networks in the visual mapping process to simultaneously increase the accuracy and speed of the mapping. Moreover, there was a study conducted on visual exploration for a mobile robot equipped with Jetson Nano [23]. That study used transformer-based networks and showed the feasibility of on-device navigation (where such a task runs on a resource-constrained device). In [24], the authors proposed a lane-detection method using hybrid convolutional and recurrent networks that smoothly run on Jeston NX. Although the method obtains higher accuracy than the compared methods, the inference speed (i.e., under 7 FPS) deteriorates. Such a computational load could be mitigated by using TensorRT-based optimization of the neural networks.

In addition, there have been benchmark studies on Jetsons for robotic applications. For instance, a benchmark result evaluates visual–inertial odometry algorithms on drones equipped with Jetson boards [25]. This study analyzes the feasibility of several algorithms on Jetson TX2, NX, and AGX with respect to accuracy and resource consumption. Additionally, it releases a novel visual–inertial odometry dataset that works successfully on Jetson devices. Some studies assess the Jetson series for deep learning applications. In [11], the authors evaluated an image classification algorithm on embedded devices, including Jetson Nano, TX2, and Raspberry Pi. The paper selected a single convolution neural network (CNN) model and evaluated the embedded devices using the model in terms of resource consumption. Moreover, there is a benchmark that tests Jetson Nano and TX2 for various CNN-based image classifications [12]. Another work in [13] adopted point cloud classification for the benchmark. In this study, the authors selected two deep learning-based models and analyzed their computational loads on Jetson Nano, TX1, and AGX. However, real-time 3D object detection, which is a fundamental task for autonomous driving, has not been clearly tested on Jetson devices.

In this work, we benchmarked various deep learning-based 3D object detectors on Jetson boards. Point cloud data processing for object detection usually demands time-consuming resource consumption due to the massive data sizes. In addition, consecutive post-processing for constructing 3D bounding boxes of detected objects poses a significant computational challenge. We aim to analyze the performance of such complex point cloud processing on Jetson boards. Additionally, we evaluate the effect of the TRT library on enabling faster inference with fewer resource usage by optimizing the neural network models of the 3D detection frameworks. We provide experimental results in terms of detection accuracy, FPS, and computational resource usage with power consumption.

## 3. Preliminaries

This section presents the preliminaries of our study. First, we describe the specifications of the four Jetson platforms. Additionally, we compare the inference performances, measured in terms of average precision (AP), of the 3D object detection frameworks that are the targets of our benchmark.

### 3.1. NVIDIA Jetson Platforms

There are four commercially available Jetson series, including Nano, TX2, NX, and AGX. Among those boards, Nano and AGX contain the lowest computing resources and the highest resources, respectively. Table 1 shows the detailed specifications of the four platforms. A description of the four platforms is as follows:

- Nano: This single board offers the opportunity to run simple deep-learning models by using a built-in GPU. However, it may suffer from the lack of memory resources when used for large-scale deep learning models.
- TX2: Jetson TX2 has been widely used for deep learning-based robotic applications owing to its better resources than those of Nano.
- Xavier NX: NX provides sufficient resources for deploying large-scale deep learning models. Moreover, NX is beneficial for various robotic systems with small payloads due to its light size and weight.
- AGX Xavier: This includes the most powerful hardware among the Jetson series. It could be suitable for industrial robots and drones that require many software functions for a mission.

### 3.2. Precision Analysis of Deep Learning-Based 3D Object Detection Frameworks

**Figure 2:**
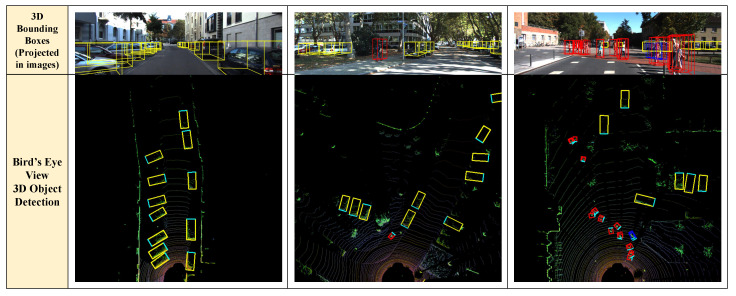
Examples of 3D object detection; the first row illustrates projection results where BEV-based detected bounding boxes are projected in images; the second row shows BEV-based 3D object detection results. In the second row of images, the yellow, red, and blue bounding boxes mean the detected car, pedestrian, and bicycle, respectively.

All the frameworks compared in this study employ point cloud data collected by a LiDAR sensor to detect the 3D positions of objects using a deep learning-based detection scheme. Table 2 shows a list of the compared frameworks (detectors), and all of these methods are available as open-source software on their respective GitHub pages. We also evaluate the detection accuracy (i.e., AP) of the detectors using the Xavier AGX board. Since the accuracy values of deep learning models are invariant for the same datasets and parameters, we only measure AP with the AGX platform. We adopt a validation set (3769 samples) on the KITTI dataset with BEV and 3D evaluation modes [14]. We evaluate AP for the car class with a moderate detection level and 0.7 intersection-over-union (IoU) setting. Figure 2 shows detection examples using one of the 3D object detection frameworks [26]. The images in the first row illustrate the 3D object detection results in which detected bounding boxes from the bird’s eye view (BEV) are combined with monocular images for visualization. The second row shows BEV-based 3D object detection results.

Table 3 shows the performance comparison among the state-of-the-art 3D detectors that we use for the benchmark (we exclude CenterPoint [19] in the table since the open-source framework exploits the nuScenes dataset [27], not the KITTI dataset. We measure the performance of the algorithm with the corresponding TRT-based version [28] in Section 4.5). We observed that the precision of one-stage detectors almost approaches that of two-stage detectors. In particular, SE-SSD [9] shows the best accuracy for car detection in the bird’s eye view (BEV) mode. In the next section, we will measure the inference speed and resource usage of the Jetson platforms for the compared frameworks.

**Table 2:** Compared frameworks: one-stage and two-stage detectors.

One-Stage Detector	Two-Stage Detector
Complex-YOLOv3 w/Tiny version [29]	PointRCNN [3]
Complex-YOLOv4 w/Tiny version [26]	Part-$A^{2}$ [5]
SECOND [7]	PV-RCNN [4]
PointPillar [21]	CenterPoint w/TRT version [28]
CIA-SSD [9]	
SE-SSD [10]	

## 4. Benchmark Analysis

In this section, we present extensive benchmark results of the Jetson platforms that run deep learning-based 3D object detectors. During the benchmark, we measure the following three metrics: (i) FPS for computational complexity measurement; (ii) resource usage, including CPU, memory, and GPU; (iii) power consumption.

### 4.1. Setup

#### 4.1.1. Environmental Setup

To thoroughly measure the performance of the detectors, we set the Jetson platforms to maximum CPU usage mode, which uses all the CPU cores with the maximum frequency and consumes maximum power (the maximum performances of the platforms are describe in Section 3.1). From this setting, we correctly measure the best performance of each platform for the 3D detection tasks (the Jetson platforms share memory resources with the GPU). For the benchmark, the platforms only run 3D object detectors without any other software so that the best performance can be measured. Every benchmark starts from the idle status of the Jetson platform. In addition, we use the following Software settings: (i) We set all Jetson platforms with Ubuntu 18.04, JetPack 4.4.1, and CUDA 10.2. (ii) All platforms use PyTorch 1.6 [30] and TRT library 7.1.3 [15]. (iii) We exploit the Jetson stats library [31] to obtain resource usage and power consumption values of the Jetson platforms.

#### 4.1.2. Default Setting for the Frameworks

As described in Section 3.2, we adopted the validation set of the KITTI dataset for the whole benchmark process. To measure the optimal performance of the frameworks, we follow the recommended parameter values of each detector for the benchmark (the default parameter settings (i.e., cfg file) can be founded on the following GitHub page, https://github.com/LoyalLumber/Benchmark_3DOD accessed on 14 March 2023). In order to check the effect of an optimization scheme, we select an open-source framework (CenterPoint [28]) that modifies inference steps of the original version [19] using TRT [15]. We also adopt a mini set of nuScenes dataset [27] that is built with a 32-channel LiDAR to compare the performance changes between the original version with PyTorch and the modified version with TRT.

### 4.2. Feasibility Check of the 3D Detectors

First, we check the feasibility of the compared frameworks to determine whether each Jetson device can handle such complex algorithms. Table 4 shows the operation test results. We observe that Jetson AGX Xavier can smoothly handle all detectors. However, the other platforms are unable to run the entire frameworks due to resource limitations. Jetson Nano and TX2 show an out-of-memory error of the GPU (i.e., CUDA error) when operating some detectors. Additionally, the NX fails to run CenterPoint due to a shortage of memory resources. However, we confirm that the Complex-YOLO variations and the TRT-based CenterPoint can be deployed on all Jetson platforms.

### 4.3. Analysis of Computational Speed

**Figure 3:**
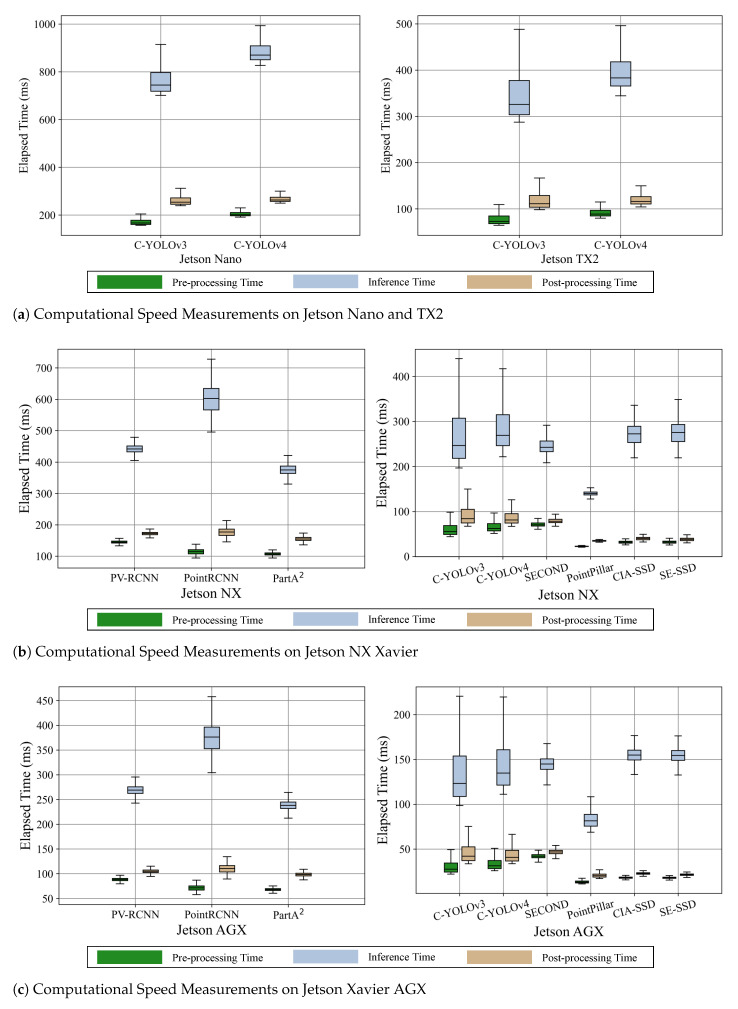
The evaluation results of the computational speeds on the four platforms. Note that Jetson Nano and TX2 are only available to run C-YOLOv3 and C-YOLOv4 frameworks due to resource limitations. TX2 shows almost double the performance compared to Nano when operating C-YOLOv3 and v4. Xavier AGX provides around 1.6 times faster computation than NX for running overall frameworks.

Figure 3 represents the result of measuring computational speed including three subdivided steps (e.g., pre-processing, inference, and post-processing). In Table 5, we present an FPS metric considering such whole steps (i.e., end-to-end) of deep learning inference.

As shown in Figure 3, the inference step using GPU resources takes the most time, and the post-processing step requires more computations than those of post-processing (both pre-processing and post-processing usually utilize CPU resources for the computation). Although the pre-processing involves encoding the LiDAR point cloud, post-processing demands larger computations during non-maximum-suppression (NMS) in which overlapping bounding boxes of objects are merged. As shown in Figure 3b,c, we notice that the two-stage detectors require more computations for pre-processing than the one-stage detectors since the two-stage algorithms usually include heavier prior data processing such as reshaping and proposal stage [3,4,5]. When comparing the NX and AGX platforms, we observe that AGX not only takes almost double the time during inference but also during pre-processing and post-processing steps. This indicates that the AGX has much more powerful resources in terms of both CPU and GPU. From these observations, we find that the pre-processing and post-processing steps require as much time as the inference step. Thus, it is essential to optimize both pre-processing and post-processing steps to reduce the overall computation time and improve the performance of the object detection system.

As identified in Table 5, we confirm that the one-stage detectors are much faster than the two-stage detectors overall, with FPS values doubled. Although the two-stage detectors assure high precision of the 3D object detection, these frameworks achieve only around 2 FPS even with the AGX platform due to the complex refinement stage. With such a low inference speed, it can be hard to support safe navigation or tasks for robotic platforms.

The end-to-end measurement results show that the AGX platform obtains the fastest inference (29.2) with Complex-YOLOv3-tiny, which almost satisfies real-time inference with 30 FPS. However, the tiny variations of Complex-YOLO have limited detection accuracy due to the compressed size of neural network models. Based on both AP and FPS, PointPillar could be the suitable choice among the compared frameworks. PointPillar achieves almost 6 and 10 FPS on the NX and AGX platforms, respectively. Moreover, the framework has a comparable AP with that of the two-stage detectors (0.871 shown in Table 3).

### 4.4. Analysis of Resource Usage and Power Consumption

**Figure 4:**
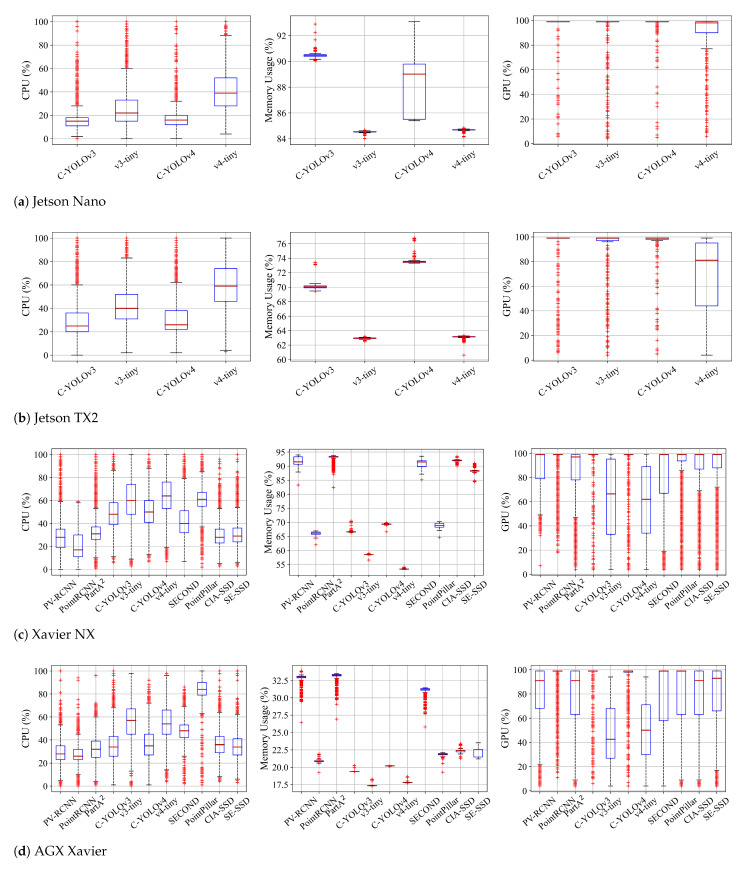
Statistical comparison of CPU, memory, and GPU usage for feasible detector–platform combinations. Nano and TX2 platforms are only available to run the Complex-YOLO frameworks.

We analyze resource usage in terms of CPU, memory, and GPU. The results are shown in Figure 4. As identified in Section 4.2, Nano and TX2 can only run the Complex-YOLO frameworks [26,29] due to insufficient memory.

Considering the Nano and TX2 platforms, the vanilla versions of Complex-YOLO (v3 and v4) tend to consume fewer CPU and more memory resources compared to the tiny versions. Such frameworks demand more memory and GPU resources due to the larger size of neural network models. We notice that Complex-YOLOv4-tiny is loosely bounded and shows higher CPU consumption than GPU usage compared to Complex-YOLOv3-tiny, which continuously requires high GPU resources. The v4-tiny sometimes exploits CPU resources with the full load (100% usage) when operated by TX2. We conjecture that the framework is not yet optimized to use GPU resources and consider it has the potential to increase FPS by fully using GPU.

In terms of resource usage on NX and AGX, both types of detectors (two-stage and one-stage) fully utilize GPU, as shown in the rightmost figures of Figure 4c,d. We also observe that the two-stage detectors (PV-RCNN, PointRCNN, and Part-$A^{2}$) require less CPU resources compared to one-stage detectors, with CPU usage being less than 60%. AGX shows significantly lower memory usage compared to other Jetson platforms due to its larger memory capacity of 32 GB. However, Xavier NX may suffer from insufficient memory when operating some of the two-stage and one-stage detectors (SECOND, CIA-SSD, and SE-SSD), as these detectors tend to consume more than 90% of memory resources. Considering the overall results of FPS and resource usage, the Complex-YOLO series and PointPillar would be preferable solutions among the compared detectors since they reserve resources to enable other functions or tasks for robotic platforms.

Table 6 shows the measurements of power consumption. In Nano and TX2, the vanilla versions of Complex-YOLO consume more power than the tiny versions since the vanilla versions require more memory resources due to the larger sizes of neural networks. Such larger models perform GPU-based complex matrix computations. In NX and AGX, we observe that one-stage detectors demand more power than two-stage detectors. PointPillar shows the highest power consumption on both platforms. Especially, AGX consumes around 29W while it runs PointPillar since the detector utilizes CPU more than 60%.

### 4.5. Analysis of the Effect of TensorRT Library

We validate the effect of the TRT library by comparing CenterPoint [19] and CenterPoint-TRT [28]. Note that Nano, TX2, and NX cannot run CenterPoint due to the out-of-memory problem. First, we measure the AP of the two frameworks for car detection. With the mini set of the nuScenes dataset, the original CenterPoint and the TRT-optimized version show mean AP of 0.88 and 0.87, respectively.

**Figure 5:**
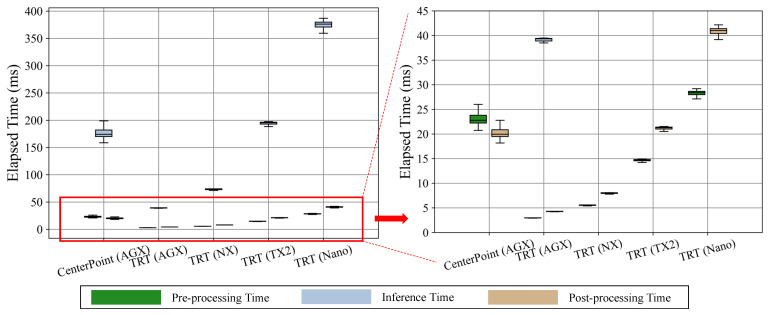
The computational time comparisons when deploying CenterPoint and CenterPoint-TRT models. From the AGX platform, we observe that CenterPoint-TRT shows an almost four times faster inference than that of the CenterPoint framework.

The corresponding computational time measurements are shown in Figure 5. Comparing the normal version of CenterPoint (CenterPoint(AGX)) and the TRT version (TRT(AGX)), we observe that the TRT library can effectively reduce a significant amount of time during the inference process. The inference time is reduced to almost one-third of its original level. In addition, the TRT library effectively reduces the time required for CPU-dominant pre-processing and post-processing steps for all platforms. We notice that the computational time is reduced by approximately two times with each improvement of the platform’s hardware specifications.

From end-to-end FPS measurements as shown in Table 7, we verify that the TRT library significantly boosts the inference speed where CenterPoint-TRT shows around a four times faster inference (18.4 FPS) than that of CenterPoint (4.41 FPS) on the AGX platform. Although the TRT version has almost the same AP as the original CenterPoint, the TRT variation achieves much faster inference. This promising result confirms that the TRT library [15] can successfully optimize a deep learning model of a 3D detector to enable faster inference without much loss of AP.

**Figure 6:**
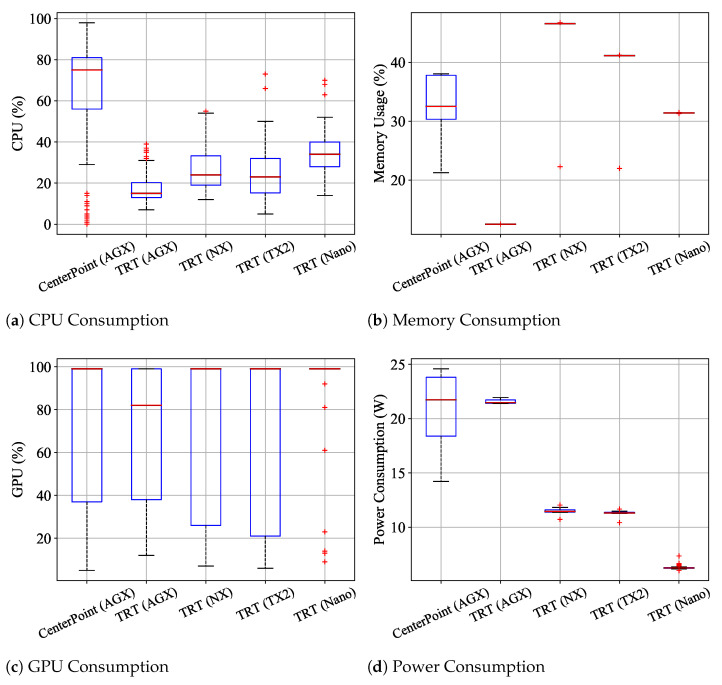
The benchmark results of CenterPoint and CenterPoint-TRT on the four platforms.

Figure 6 and Table 8 show the measurements of resource usage and power consumption for the two frameworks. In terms of resource usage on the AGX platform, CenterPoint consumes more resources (including CPU, GPU, and memory) than the TRT version. This result indicates that the TRT library not only increases the inference performance (i.e., FPS) but also enables more efficient resource consumption for the Jetson platforms. Moreover, all platforms tend to reserve many CPU and memory resources when operating CenterPoint-TRT, with NX and AGX platforms utilizing only around 27% and 18% of CPU resources, respectively. With such resource margins, Jetson-based robotic systems can simultaneously operate a 3D object detector and navigation tasks, including path planning, localization, and controls. In terms of power consumption on the AGX platform, CenterPoint-TRT shows more stable power consumption than CenterPoint, with a relatively small standard deviation (0.19). We confirm that the TRT library could be essential for Jetson-based robotic systems that require running many functions in real time.

## 5. Conclusions

In this work, we examined deep learning-based 3D object detection frameworks on all commercially available Jetson platforms (i.e., Nano, TX2, NX, and AGX). Our objective was to analyze Jetson platforms in terms of detection accuracy, FPS, and resource usage when operating complex point cloud processing that demands high computational resources. We selected the state-of-the-art 3D detectors within two categories (one-stage and two-stage). From the experiments, it was observed that Nano and TX2 are unable to handle complex computations of the two-stage detectors and most of the one-stage detectors due to the shortage of memory resources. Otherwise, it was found that all Jetson platforms successfully run the Complex-YOLO series. For NX and AGX platforms, PointPillar would be suitable since it provides high AP and fast inference with stable resource usage. We observed that all of the platforms, on average, consume 50% and 80% of the resources of CPU and GPU, respectively. Remarkably, Jetson AGX only requires less than 32% of RAM resources for the entire compared algorithms. In addition, we confirm that the TRT library [15] not only maintains detection accuracy but also significantly increases the FPS of a 3D detector. When processing CenterPoint-TRT, the AGX consumes only half of the CPU and memory compared to the original CenterPoint algorithm. TRT has the potential to reserve a significant amount of CPU and memory resources for Jetson platforms, allowing them to simultaneously operate a 3D detector with other navigation functions for robotic applications. In the future, we plan to apply an optimization approach to the compared 3D object detection frameworks to boost the performance of the Jetson series. To accomplish this, we will adopt the TRT library, which automatically tunes functions of deep neural networks. We will provide extensive benchmark tests with various TRT-based frameworks on the Jetson platforms. 

## Figures and Tables

**Table 1:** Specification of the four Jetson platforms.

	Nano	TX2	NX	AGX
**AI** **Core**	472 GFLOPs	1.33 TFLOPs	21 TOPs	32 TOPs
**CPU**	4-core Cortex A57	6-core Denver A57	6-core Carmel Arm	8-core Carmel Arm
**GPU**	128-core Maxwell	256-core Pascal	384-core Volta	512-core Volta
**Memory**	4 GB 64-bit LPDDR4	8 GB 128-bit LPDDR4	8 GB 128-bit LPDDR4	32 GB 256-bit LPDDR4
**Size** **(mm)**	100 × 80 × 29	50 × 110 × 37	100 × 90 × 32	105 × 105 × 65
**Power**	5 W (or 10 W)	7.5 W (or 15 W)	10W (or 15, 30 W)	10 W (or 15, 30 W)
**Weight**	100 g	211 g	184.5 g	670 g

**Table 3:** Comparison of AP (IoU = 0.7).

Type	Method	Car—AP (BEV)	Car—AP (3D)
**Two-stage** **Detector**	PointRCNN	0.878	0.784
	Part-$A^{2}$	0.886	0.794
	PV-RCNN	0.879	0.836
**One-stage** **Detector**	Complex-YOLOv3 (Tiny version)	0.82 (0.673)	-
	Complex-YOLOv4 (Tiny version)	0.833 (0.68)	-
	SECOND	0.837	0.756
	PointPillar	0.871	0.772
	CIA-SSD	0.867	0.761
	SE-SSD	0.883	0.792

**Table 4:** Feasibility check of the compared detectors. In the table, the check mark symbol (✓) indicates that the hardware platform can successfully run a detector, while the X symbol indicates that it fails to do so due to resource constraints. Based on our analysis, we find that both Nano and TX2 do not have sufficient computation resources to operate most one-stage detectors and all two-stage detectors.

Type	Method	Nano	TX2	NX	AGX
**Two-stage** **Detector**	PointRCNN	X	X	✓	✓
	Part-$A^{2}$	X	X	✓	✓
	PV-RCNN	X	X	✓	✓
	CenterPoint (TRT version)	X (✓)	X (✓)	X (✓)	✓ (✓)
**One-stage** **Detector**	Complex-YOLOv3 (Tiny version)	✓ (✓)	✓ (✓)	✓ (✓)	✓ (✓)
	Complex-YOLOv4 (Tiny version)	✓ (✓)	✓ (✓)	✓ (✓)	✓ (✓)
	SECOND	X	X	✓	✓
	PointPillar	X	X	✓	✓
	CIA-SSD	X	X	✓	✓
	SE-SSD	X	X	✓	✓

**Table 5:** Average FPS (i.e., end-to-end elapsed time for an inference) of the compared detectors.

Type	Method	Nano	TX2	NX	AGX
**Two-stage** **Detector**	PointRCNN	-	-	1.2	1.98
	Part-$A^{2}$	-	-	1.82	2.54
	PV-RCNN	-	-	1.43	2.27
**One-stage** **Detector**	Complex-YOLOv3 (Tiny version)	1.6 (3.5)	2.2 (7.3)	2.95 (17.93)	5.13 (29.2)
	Complex-YOLOv4 (Tiny version)	1.5 (7)	2.3 (12.1)	2.82 (16.4)	5.05 (26.7)
	SECOND	-	-	2.6	5.21
	PointPillar	-	-	5.73	9.7
	CIA-SSD	-	-	3.12	5.79
	SE-SSD	-	-	3.17	5.82

**Table 6:** Measurements of average power consumption (W). The table presents mean values with standard deviations.

Type	Method	Nano	TX2	NX	AGX
**Two-stage** **Detector**	PointRCNN	-	-	11.4±0.4	23.3±0.8
	Part-$A^{2}$	-	-	11.8±0.6	23.5±1.2
	PV-RCNN	-	-	11.6±0.5	23.3±0.9
**One-stage** **Detector**	Complex-YOLOv3 (Tiny version)	6.4±0.5 (5.5±0.4)	13.1±0.4 (9.9±0.5)	12.6±0.6 (8.5±0.4)	26.1±1.1 (17.1±0.7)
	Complex-YOLOv4 (Tiny version)	6.6±0.3 (5.5±0.4)	13.7±0.7 (9.3±0.7)	13.2±0.6 (8.9±0.4)	27.6±1.3 (17.8±0.5)
	SECOND		-	12.7±0.7	26.7±1.4
	PointPillar	-	-	13.8±1	29.1±2.3
	CIA-SSD	-	-	12.3±0.8	24.8±1.7
	SE-SSD	-	-	12.4±0.7	24.7±1.7

**Table 7:** Averaged end-to-end FPS (Hz) of CenterPoint and CenterPoint-TRT. Most Jetson platforms, except AGX Xavier, cannot run the original CenterPoint framework. On the other hand, all platforms can handle the TRT version of CenterPoint owing to the efficient computation strategy from the TRT library.

Method	Nano	TX2	NX	AGX
CenterPoint	-	-	-	4.41
CenterPoint-TRT	1.71	4.29	10.43	18.4

**Table 8:** Measurements of resource usage and power consumption for CenterPoint and CenterPoint-TRT. The table provides mean values with standard deviations.

Method	Resource	Nano	TX2	NX	AGX
**CenterPoint**	CPU (%)	-	-	-	63.3±26.9
	Mem (%)	-	-	-	33.3±4.7
	GPU (%)	-	-	-	76.1±34.6
	Power (W)	-	-	-	20.9±3.1
**CenterPoint-TRT**	CPU (%)	33.9±9.1	24.9±12.3	26.5±9.9	17.7±7.46
	Mem (%)	62.8±0.1	40.1±4.4	45.1±6.1	12.5±0.1
	GPU (%)	86.9±28.9	70±42.7	70.5±42.5	66.7±34.5
	Power (W)	6.3±0.2	11.3±0.2	11.5±0.3	21.5±0.2

## Data Availability

Not applicable.

## Abbreviations

The following abbreviations are used in this manuscript:

MDPI	Multidisciplinary Digital Publishing Institute
DOAJ	Directory of Open Access Journals
TLA	three-letter acronym
LD	linear dichroism
